# Media Exposure and Substance Use Increase during COVID-19

**DOI:** 10.3390/ijerph18126318

**Published:** 2021-06-11

**Authors:** Ofer Amram, Porismita Borah, Deepika Kubsad, Sterling M. McPherson

**Affiliations:** 1 Department of Nutrition and Exercise Physiology, Elson S. Floyd College of Medicine, Washington State University, Spokane, WA 99202, USA; 2 Paul G. Allen School for Global Animal Health, Washington State University, Pullman, WA 99164, USA; 3 Edward R. Murrow College of Communication, Washington State University, Pullman, WA 99163, USA; p.borah@wsu.edu; 4 School of Medicine, University of Washington, Seattle, WA 98195, USA; deepika.kubsad@wsu.edu (D.K.); smcpherson05@wsu.edu (S.M.M.); 5 Department of Community and Behavioral Health, Elson S. Floyd College of Medicine, Washington State University, Spokane, WA 99202, USA; 6 Analytics and PsychoPharmacology Laboratory, Washington State University, Spokane, WA 99202, USA; 7 Program for Excellence in Addictions Research, Washington State University, Spokane, WA 99202, USA

**Keywords:** COVID-19, substance abuse, drug misuse

## Abstract

Background: Lockdown measures because of COVID-19 are likely to result in deteriorating physical and mental health. In this study, our aim was to assess the impact of media exposure on increases in substance use during the COVID-19 pandemic. Methods: A nationally representative online survey of 1264 adults was collected during the pandemic in the United States. Logistic regression was used to explore the association between an increase in substance use since the beginning of the COVID-19 pandemic and exposure to cable news or social media together with COVID-19 knowledge, while controlling for covariates. Results: In the multivariable-adjusted models, participants with the highest exposure to social media (at least daily) and low knowledge of COVID-19 were 9.9 times more likely to experience an increase in substance use since the pandemic began (OR = 9.90, 95% CI = 4.27–23.06). Participants with the highest exposure to cable news and low knowledge of COVID-19 were over 11 times more likely to experience an increase in substance use (OR = 11.64, 95% CI = 4.01–24.45). Conclusion: Based on our findings, we recommend that media organizations should aim to reduce uncertainty and also provide positive coverage to counter the negative information associated with pandemics.

## 1. Introduction

Social distancing and lockdown measures as a result of COVID-19 have had a negative impact on the physical and mental health for many groups in society, particularly those who are vulnerable and at high risk regardless of the pandemic [1,2]. Individuals who had mental health concerns and needs prior to COVID-19, including anxiety and depression, were likely to experience an increase of their symptoms [3].

Prior research has shown that media exposure is associated with increased distress, anxiety and substance use [4,5,6,7,8,9]. Increased audiovisual media, television, and social media have a greater impact on users’ mental health [6,10,11]. Many studies have shown the association between television news use and negative emotional reactions [12,13]. A recent study has provided evidence that higher use of social media news was associated with higher anxiety in participants in China during the COVID-19 pandemic [6]. Similar findings have been demonstrated in the U.S. [4]. However, other recent research has shown that media exposure in general increased fear and worry about COVID-19 in participants, with television and online media causing the most distress [5]. To date, no known studies have investigated the impact of media exposure during COVID-19 on substance use.

There has been evidence suggesting that drug overdose events have been rising during the COVID-19 pandemic [14]. To date, six federal agencies in the United States, the National Institute on Drug Abuse, Substance Abuse and Mental Health Services administration, Centers for Medicare & Medicaid Services, Food and Drug Administration, Centers for Disease Control, and Office of National Drug Control Policy, declared that opioid misuse was a national epidemic [15,16]. As the pandemic continues, substance use remains a growing public health concern, and that concern grows exponentially just as the negative health outcomes grow exponentially with the use of more than one substance. Using a national survey from across the United States, our aim was to assess the impact of media exposure on increases in substance use during the COVID-19 pandemic. More specifically, we assessed the frequency of use of both cable news and social media, the two-communication media that are the primary sources of knowledge and information about COVID-19. Our hypothesis was that increased exposure to media would be associated with increased substance use and number of substances used.

## 2. Materials and Methods

A nationally representative online survey of 1264 adults over 18 years of age collected responses from June 22 to July 18, during the peak of the pandemic in the United States. At that time, most states were in some form of lockdown status and were the theater of several lockdown- and social-justice-related demonstrations. This national Qualtrics panel included an over-sample of Washington state residents for a different study (*N* = 416) and employed demographic and regional quotas based on the 2019 U.S. census. Quality check measures eliminated duplicate responses and responders who answered in patterns, provided illogical patterns of answers, or sped through the survey in less than a third of the overall median survey duration. In addition, post-stratified sample weights were applied to adjust for the Washington State oversample and to ensure that the quota samples from the four U.S. regions reflected 2019 census estimates [17,18]. This study was approved by the Institutional Review Board of Washington State University.

### 2.1. Measures

Outcome variable: The outcome variable for this study was whether a participant experienced an increase in substance use since the beginning of the COVID-19 pandemic (yes vs. no). This referred to four categories of drugs including prescription drugs, non-prescription drugs, sedatives/hypnotics, and methamphetamines (i.e., four different questions, one for each drug). Those drugs were chosen because of their incidence of long-term dependencies.

Exposure variable: The primary aim was to assess whether exposure to media (cable news or social media) and COVID-19 knowledge could explain the increase in substance use noted during the pandemic. Therefore, we first created two different media exposure measures, one for cable news and one for social media. Participants were asked about the frequency of use of different media outlets, and the variable was categorized into three levels using the following criteria: no use or monthly use of media was categorized as low, weekly use of media as medium, and at least once a day as high. The following cable news media outlets were combined into one measure of cable news exposure: CNN, Fox news, and MSNBC. The same measure was created to assess exposure to social media, combining four questions about social media resources that included getting information from social media friends, influencers, community members, and what was currently trending (e.g., hashtags). The primary explanatory variable was an interaction variable with COVID-19 knowledge and each of the media exposure levels. Regarding knowledge of COVID-19, participants rated a series of statements as true or false, with correct answers totaled, based on information from the World Health Organization (2020), the Centers for Disease Control (2020), and a contemporary Research Triangle Institute (RTI), (2020); survey COVID-19 knowledge was dichotomized to high (at least four correct answers) and low knowledge (less than four correct answers) (Table 1). We used the knowledge variable together with the media exposure variable as it acted as a moderator of substance use. More specifically, we could expect individuals with high knowledge of COVID-19 to be less impacted by exposure to either cable news or social media. This is because, in general, individuals with higher knowledge will have had lower uncertainty about the pandemic, and as a result, they will have been less impacted by the media exposure [9]. Each of the primary explanatory variable had six levels of interaction between knowledge and media exposure: Low knowledge—Low media exposure, Low knowledge—Mid media exposure, Low knowledge—High media exposure, Mid knowledge—Low media exposure, Mid knowledge—Mid media exposure, and Mid knowledge—High media exposure.

Covariates: The following variables were included in the model as covariates: Education level (high school graduate vs. non-high school graduate), ethnicity (white vs. nonwhite), age (18–39, 40–59, and 60 or over (categorical)), gender (male vs. female), income ($0–$49,999, $50,000–$74,999, and above $75,000) and political orientation (liberal vs. moderate vs. conservative). We also included a measure of rurality based on the participant’s zip code. We used the Rural Urban Commuting Area (RUCA) to classify the participant’s zip code place of residence based on their degree of rurality, using the following: urban, large rural, small rural, and isolated zip code.

### 2.2. Analysis

Univariate analyses included the reporting measure of central tendency and variability for continuous variables and frequency distributions and percentages for categorical variables. Bivariate statistics included chi-square and the Mann–Whitney U to test for differences in demographic and exposure variables in the outcome variable groups. Multivariate analyses included generalized linear models (GLMs) with a binary logistic function to explore the association between media exposure and substance use increase during COVID-19, controlling for covariates. Associations were presented as odds ratios (ORs) with 95% confidence intervals (CIs). R was used to perform statistical modeling procedures, using a significance level of <0.05.

## 3. Results

The characteristics of individuals who experienced an increase in substance use are shown in Table 2. A total of 1264 participants completed the panel survey; 51 were dropped because they did not provide information on the outcome variable, for a total of 1213 participants to be included in the analysis. Of those, 197 (16.7%) participants experienced a substance use increase; 772 (63.3%) were white, and 592 (48.8%) were male; 234 (19.3%) were over the age of 60; 475 (39.2%) described themselves as liberal (as opposed to 35.9% conservative and 18.5% moderate); 300 (24.7%) earned less than $50,000 a year, and 265 (21.8%) had only a high school degree. Geographically, 76.4% of the participants in the survey resided in urban zip codes, closely matching the actual rural/urban distribution in the US Census (United States Census Bureau, 2016) [19].

**Figure 1:**
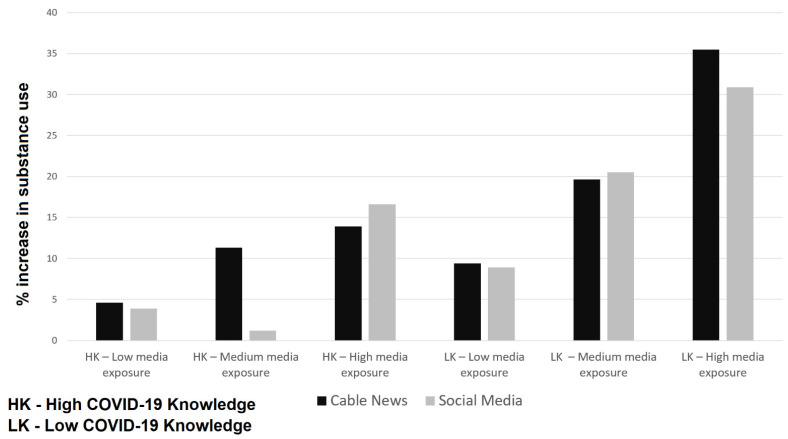
Increases in substance use during COVID-19 and the two primary explanatory interaction variables representing COVID-19 knowledge and media exposure (cable news and social media).

Finally, 197 (16.2%) reported an increase in substance use during COVID-19, and of those, only 6.4% were over the age of 60 (compared to 21.4% for ages 40–59 and 17% for ages 18–39), 18.4% were male and 13.9% were female, 22.9% earned $75,000 or more (compared to 10.7% for those who earned $50,000–$75,000 and 15.3% of those who earned less than $50,000), and 21.6% self-described as conservatives (compared to 15.9% liberal and 13% moderate). A total of 35.5% of participants with both low knowledge and high exposure experienced an increase in substance use, while only 4.6% of those with high knowledge and low exposure experienced an increase. The same trend was also observed for exposure to social media and COVID-19 knowledge; participants with both low knowledge and high exposure experienced the highest increase in substance use (30.9%), while those with high knowledge and low exposure only had a 3.9% increase (Figure 1).

In the multivariable-adjusted models, an increase in substance use during COVID-19 was associated with low knowledge of COVID-19 and increased exposure to both social media and cable news (Table 3). More specifically, in the social media adjusted model, participants with the highest exposure to social media (at least daily) and low knowledge of COVID-19 were 9.9 times more likely to experience an increase in substance use since the pandemic began (OR = 9.90, 95% CI = 4.71–23.61). This exact same trend was also observed in the cable news model, and participants with the highest exposure to cable news and low knowledge of COVID-19 were over 11 times more likely to experience an increase in substance use (OR = 11.64, 95% CI = 4.01–24.45). In addition, participants age 40–59 experienced much higher substance use in both the cable news and social media models (OR = 1.49, 95% CI = 1.02–2.1 and OR = 1.68, 95% CI = 1.15–2.47, respectively), and participants 60 years and older experienced lower substance use only in the cable news model (OR = 0.51, 95% CI = 0.27–0.93). Finally, male participants experienced a notable increase in substance use only in the social media model (OR = 1.49, 95% CI = 1.02–2.19).

## 4. Discussion

The findings of this study showed that increased exposure to both cable news and social media were associated with substantial increases in substance use during the COVID-19 pandemic. Interestingly, older populations (60 years old and over) experienced decreased odds of substance use when exposed to cable news, while younger populations (ages 40–59) experienced a higher likelihood of substance use with exposure to both cable news and social media. To date, no known studies have investigated the impact of media exposure during COVID-19 on substance use.

Several studies have shown a clear relationship between media exposure (either television or social media) during COVID-19 and increased distress and anxiety [4,6,12]. The results presented here confirm these studies by showing that increased media exposure not only increases anxiety, but is also likely to increase substance use. Since COVID-19 emerged in early 2020, there has been a tremendous demand for information about the pandemic. Social media platforms have been used to disseminate information and stories on COVID-19 by organizations and individuals. Some of this information has been misleading and unfounded, leading to fear and confusion, which in turn has increased anxiety and distress. Constant exposure to cable news, which tends to have better control over the content presented than social media, was also shown to induce anxiety and distress during COVID-19 and other public health crises. As a highly visual platform, increased exposure to television can also cause viewer fear and mental distress [10]. The findings in the study presented here also indicate that increased knowledge of COVID-19 could moderate the impact of media exposure and could decrease the likelihood of substance use. This is critically important, as this allows individuals to better navigate the influx of information on COVID-19, and it could act as a tool to decrease uncertainty about COVID-19 information and, in turn, reduce the likelihood of substance use.

Interestingly, while our results showed that those between 40 and 59 years of age were more likely to experience increased substance use when exposed to both cable news and social media, it also showed that those over 60 were less likely to experience substance use when exposed to cable news. This may be because mental-health problems, like depression, occur less frequently within older populations, which may, in turn, indicate a decreased likelihood of substance use [19]. It may also be because older populations are more likely to consume news from cable TV than social media and that cable news has a less detrimental impact on mental health [20,21]. Additional research is needed to better understand the differences in the results between these age groups.

Increased dependency on illicit substance use remains a rising concern for both individuals with preexisting anxiety, stress, and substance use disorders (SUDs) and among individuals previously free of these conditions [22]. The psychosocial impact of COVID-19 has continued to act as a severe stressor and impact the mental and general health outcomes within the public. It is especially concerning that after prolonged exposure to stress, substance use and their consequential neurobiological changes continue to persist even after the stressor is removed, further perpetuating the cycle of substance use [23,24]. Substance use carries a high risk of pulmonary and cardiovascular comorbidities, with complications due to the immunosuppressive qualities linked to the use of alcohol, opioids, and stimulants [25]. The ongoing pandemic is exasperating the existing complications of treating substance and opioid use for clinicians and patients, due to the decreased access to treatment for those with substance use disorders. Thus, it remains imperative that clinicians have accessible information and guidelines to care for these vulnerable individuals during this unprecedented time and that they remain vigilant in prescribing opioid medication.

### Limitation

An important limitation that needs to be acknowledged is that our survey was limited to respondents willing and able to participate in a Qualtrics panel survey. This is especially impactful in geographic areas with limited access to broadband, which has a greater effect on rural areas and isolated communities. However, to somewhat overcome this limitation, we made sure that the rural urban distribution of our survey participants closely matched the actual rural/urban distribution in the US Census. In addition, we were not able to capture individual distress or reasons for using substances. Our survey did not include measures to examine the motivations for media use. For example, individuals could use social media to find information about the pandemic or for recreation purposes. These different motivations may have had a specific impact on people’s substance use. Future research should examine these nuances to better understand the relationship between media use and substance use.

## 5. Conclusions

Our findings point to the critical role played by media organizations in reducing uncertainty in evolving situations, such as pandemics, and the need for positive and hopeful messaging to counter the negative information often associated with such events. Our findings also show that increased knowledge has meaningful moderating effects on substance use and leads to decreased uncertainty. Given this, we recommend that during public health crises, like the COVID-19 pandemic, information from organizations such as the World Health Organization and Centers for Disease Control be prioritized and that medical professionals play a role in directing patients away from high stimulation media, such as 24-hour news and social media.

## Figures and Tables

**Table 1:** List of questions to determine COVID-19 knowledge.

Please indicate if it is TRUE or FALSE, for each of the following statements about the COVID-19 virus:
1. Antibiotics can be used to treat the COVID-19 virus
2. People of all ages can become infected with COVID-19
3. People of all racial and ethnic groups can become infected with the COVID-19
4. Eating garlic can lower your chances of getting infected with the COVID-19 virus.
5. Most people who are infected with the COVID-19 virus die from it.
6. Most people who are infected with the COVID-19 virus recover from it.
7. Older adults or those with compromised immune systems are at a higher risk.

**Table 2:** Characteristics of survey participants stratified by whether they experienced increased substance use during COVID-19 (*n* = 1213).

	Substance Use Increase during COVID-19			
Characteristics	*N* = 1213 (100%)	No Increase = 1016 (83.8%)	Increase = 197 (16.2%)	*p* Value
Age Group				-
18–39	616 (51.0)	511 (83)	105 (17)	<0.001
40–59	360 (29.7)	283 (78.6)	77 (21.4)	
60 and over	234 (19.3)	219 (93.6)	15 (6.4)	
NA	3 (0.2)	3 (0.3)	0 (0.0)	
Gender				
Female	604 (49.8)	520 (86.1)	84 (13.9)	0.034
Male	592 (48.8)	483 (81.6)	109 (18.4)	
NA	17 (1.4)	13 (1.2)	4 (2.0)	
Race				
White	772 (63.3)	654 (84.7)	118 (15.3)	0.232
Non-White	441 (36.4)	362 (82.1)	79 (17.9)	
NA	0 (0.0)	0 (0.0)	0 (0.0)	
Education Level				
High School Diploma or less	265 (21.8)	227 (85.7)	38 (14.3)	0.074
Higher Education	911 (75.1)	753 (82.7)	158 (17.3)	
NA	37 (3.0)	36 (3.5)	1 (0.5)	
Rurality				
Urban	919 (76.3)	756 (82.3)	163 (17.7)	0.052
Large rural	63 (5.1)	53 (84.1)	10 (15.9)	
Small rural	204 (16.5)	184 (90.2)	20 (9.8)	
Isolated	23 (1.8)	19 (82.6)	4 (17.4)	
NA	4 (0.3)	4 (0.4)	0 (0.0)	
Income				
<$49,999	300 (24.7)	254 (84.7)	46 (15.3)	<0.001
$49,999–$74,999	466 (38.4)	416 (89.3)	50 (10.7)	
>$75,000	442 (36.4)	341 (77.1)	101 (22.9)	
NA	5 (0.4)	5 (0.5)	0 (0.0)	
Political Orientation				
Liberal	475 (39.2)	402 (84.6)	73 (15.4)	0.012
Moderate	224 (18.5)	198 (88.4)	26 (11.6)	
Conservative	435 (35.9)	347 (79.8)	88 (20.2)	
COVID-19 Knowledge—Cable News Consumption				
LK–LCC	106 (8.7)	96 (90.6)	10 (9.4)	<0.001
LK–MCC	168 (13.8)	135 (80.4)	33 (19.6)	
LK–HCC	197 (16.2)	127 (64.5)	70 (35.5)	
HK–LCC	152 (12.5)	145 (95.4)	7 (4.6)	
HK–MCC	265 (21.8)	235 (88.7)	30 (11.3)	
HK–HCC	323 (26.2)	278 (86.1)	45 (13.9)	
NA	2 (0.1)	0 (0.0)	2 (1.0)	
COVID-19 Knowledge—Social Media Consumption				
LK–LSMC	90 (7.4)	82 (91.1)	8 (8.9)	<0.001
LK–MSMC	112 (9.2)	89 (79.5)	23 (20.5)	
LK–HSMC	269 (22.2)	186 (69.1)	83 (30.9)	
HK–LSMC	257 (21.2)	247 (96.1)	10 (3.9)	
HK–MSMC	170 (14.0)	149 (98.8)	21 (1.2)	
HK–HSMC	314 (25.9)	262 (83.4)	52 (16.6)	
NA	1 (0.0)	1 (0.0)	0 (0.0)	

Note—LK: low COVID-19 knowledge; HK: high COVID-19 knowledge; LCC: low cable news consumption; MCC: mid cable news consumption; HCC: high cable news consumption; LSMC: low social media consumption; MSMC: mid social media consumption; HSMC: high social media consumption.

**Table 3:** Unadjusted and adjusted GLMs analyses of COVID-19 knowledge together with media exposure and increase in substance use during the pandemic (*n* = 1213).

Variable	Unadjusted	Adjusted	
	OR	Cable News	Social Media
	(95% CI)	OR (95% CI)	OR (95% CI)
COVID-19 Knowledge—Cable News Consumption			
HK–LCC (Ref)	-	-	-
HK–MCC	2.64 (1.19–6.69) **	3.42 (1.16–7.44) *	-
HK–HCC	3.35 (1.56–8.30) **	4.56 (1.66–10.15) **	-
LK–LCC	2.15 (0.80–6.12)	2.94 (0.93–8.29)	-
LK–MCC	5.06 (2.29–12.82) ***	5.77 (2.06–13.49) **	-
LK–HCC	11.41 (5.40–28.11) ***	11.64 (4.01–24.45) ***	-
COVID-19 Knowledge—Social Media Consumption			
HK–LSMC (Ref)	-	-	-
HK–MSMC	3.48 (1.63–7.90) **	-	2.93 (1.25–7.48) *
HK–HSMC	4.90 (2.54–10.43) ***	-	4.85 (2.31–11.4) **
LK–LSMC	2.40 (0.89–6.31) *	-	2.65 (0.82–7.31)
LK–MSMC	6.38 (2.99–14.53) ***	-	5.56 (2.21–13.81) ***
LK–HSMC	11.02 (5.82–23.16) ***	-	9.91 (4.71–23.61) ***
Political Orientation			
Liberal (Ref)	-	-	-
Moderate	0.76 (0.45–1.26)	0.75 (0.44–1.25)	0.69 (0.40–1.14)
Conservative	1.32 (0.90–1. 94)	1.18 (0.80–1.74)	1.21 (0.82–1.78)
Age Group			
18–39 (Ref)	-	-	
40–59	1.33 (0.91–1.31)	1.49 (1.02–2.1) **	1.68 (1.15–2.47) *
60 and over	0.33 (0.18–0.57) ***	0.51 (0.27–0.93) **	0.78 (0.40–1.42)
Gender	-		
Female (Ref)	-	-	-
Male	1.39 (1.02–1.90) *	1.46 (0.99–2.15)	1.49 (1.02–2.19) *
Race	-		
Non-White (Ref)	-	-	
White	0.82 (0.60–1.13)	0.85 (0.58–1.27)	0.90 (0.60–1.34)
Rurality			
Isolated (Ref)	-	-	
Large rural	0.89 (0.26–3.57)	1.02 (0.23–5.51)	1.24 (0.30–6.45)
Small rural	0.51 (0.17–1.91)	0.47 (0.12–2.37)	0.57 (0.15–2.76)
Urban	1.02 (0.37–3.56)	0.99 (0.28–4.67)	1.17 (0.35–3.56)
Income			
<$49,999 (Ref)	-	-	
$49,999–$74,999	0.66 (0.43–1.02) *	0.79 (0.49–1.30)	0.75 (0.46–1.23)
>$75,000	1.63 (1.11–2.41) **	1.31 (0.81–2.14)	1.27 (0.79–2.08)
Education Level			
Higher Education (Ref)	-	-	
High School Diploma or less	0.70 (0.47–1.02) *	0.90 (0.56–1.42)	0.85 (0.53–1.32)

Notes: LK: low COVID-19 knowledge; HK: high COVID-19 knowledge; LCC: low cable news consumption; MCC: mid cable news consumption; HCC: high cable news consumption; LSMC: low social media consumption; MSMC: mid social media consumption; HSMC: high social media consumption; OR: odds ratio; *** *p* < 0.001, ** *p* < 0.01, * *p* < 0.05.

## Data Availability

The data presented in this study are available on request from the corresponding author.
